# Bacterial Diversity in Feline Conjunctiva Based on 16S rRNA Gene Sequence Analysis: A Pilot Study

**DOI:** 10.1155/2017/3710404

**Published:** 2017-11-27

**Authors:** Katarzyna Płoneczka-Janeczko, Jacek Bania, Karolina Bierowiec, Maciej Kiełbowicz, Zdzisław Kiełbowicz

**Affiliations:** ^1^Department of Epizootiology with Clinic for Birds and Exotic Animals, Faculty of Veterinary Medicine, Wrocław University of Environmental and Life Sciences, Norwida 31, 50-356 Wrocław, Poland; ^2^Department of Food Hygiene and Consumer Health Protection, Faculty of Veterinary Medicine, Wrocław University of Environmental and Life Sciences, Norwida 31, 50-356 Wrocław, Poland; ^3^Department and Clinic of Veterinary Surgery, Faculty of Veterinary Medicine, Wrocław University of Environmental and Life Sciences, Norwida 31, 50-356 Wrocław, Poland

## Abstract

Changes in the microbial populations in the conjunctival sacs of animals have traditionally been evaluated using conventional microbiology techniques. The goal of this study was to examine the suitability of a methodology which may reveal a previously unknown microbiome inhabiting feline conjunctival membranes. In the present study, we determined the microbial diversity in feline conjunctivas based on 16S rRNA gene sequence analysis. Five taxa not described earlier in veterinary ophthalmology (i.e.,* Staphylococcus caprae*,* Staphylococcus succinus, Propionibacterium acnes, Psychrobacter faecalis, *and* Bacillus subtilis*) were identified in feline conjunctivas with a high similarity (99-100%). The study demonstrates that the feline conjunctival sacs are inhabited by much more rich and diverse microbial communities than previously thought using culture-based methods. From the clinical perspective, this could suggest that other laboratory procedures (e.g., extended incubation time in the case of Actinobacteria, formerly order Actinomycetales) or a new tool like culture-independent approaches (next-generation DNA sequencing) should be taken into account.

## 1. Introduction

Conjunctivitis, keratoconjunctivitis, and corneal sequestration are common clinical problems in cats. Based on research over the last few decades, characteristics of the bacterial flora in feline conjunctival sacs show a similar composition, and the occurrence of particular species of bacteria varies by frequency of their isolation. However, Gelatt described the feline conjunctival and corneal surface as being generally colonized to a lower degree than in other domestic species [1]. Among bacteria isolated from the conjunctiva, staphylococci are the most representative group. The presence of* S. epidermidis*,* S. pseudintermedius*,* S. aureus*,* S. albus*,* S. haemolyticus*,* S. simulans*,* S. auricularis*,* S. saprophyticus*, and* S. felis* has been observed in the conjunctiva by many scientists [2–4]. A second group of frequently isolated microorganisms are hemolytic and nonhemolytic streptococci (i.e.,* S. viridans*) [2]. Previous studies based on the microbiological identification of bacteria or the sequencing of amplicons generated from microbial DNA have led to the identification of several genera in feline conjunctivas, such as* Enterococcus* spp.,* Pseudomonas* spp.* (P. aeruginosa)*,* Proteus* spp.,* Pasteurella* spp.,* Bacillus* spp., and* Micrococcus* spp. [3, 5].* Mycoplasma (M. felis*,* M. canadense*,* M. cynos*,* M. gateae*,* M. lipophilum*, and* M. hyopharyngis)* have also been considered conjunctival commensals, which in some circumstances may be involved in conjunctival pathology [6–9].* Chlamydophila felis* has been identified as an indisputable pathogen of feline conjunctiva. This Gram-negative bacterium has already been isolated from a number of feline conjunctivitis cases [10, 11]. There is also evidence that other* Chlamydia*-related microorganisms like* Chlamydophila pneumoniae* and* Neochlamydia hartmannellae* may be associated with conjunctiva [12, 13]. Investigating conjunctival infections in cats with lepromatous lesions, Fyfe et al. [14] identified* Mycobacterium* spp. to have occurred. Based on a phylogenetic analysis, a novel species in the* Mycobacterium simiae*-related group was identified [14]. On the other hand, Fox et al. [15] described* Salmonella*-associated conjunctivitis in cats.

Most of the previous research has investigated feline ocular microflora using a classical microbiology approach involving the culture and further characterization of isolates. The aim of the present study was to examine the suitability of the methodology which may disclose microbial diversity within feline conjunctivas of healthy cats and animals with conjunctivitis symptoms, using partial sequencing of the 16S rRNA gene. To the best of our knowledge, it is a frontier research in the field of veterinary ophthalmology and a preliminary study linked to our next project concerning next-generation sequencing (NGS).

## 2. Materials and Methods

Conjunctival swabs obtained from three clinically healthy cats with no ocular disorders and from three cats with conjunctivitis symptoms were included in the study. Based upon our own clinical experience with chronic conjunctivitis in cats and for the purpose of the study, sick animals comply with criteria such as manifestation of conjunctivitis (ocular discharge, chemosis, and conjunctival edema) lasting about six months and insufficient response to the standard ophthalmological treatment (history of the treatment with ophthalmic ointments and eye drops). Sick cats were also tested by PCR and RT-PCR to determine the presence of* Chlamydia felis*, feline herpesvirus-1 (FHV-1), and* Mycoplasma felis *infections, according to published protocols by Chalker et al. [16], Marsilio et al. [17], and Helps et al. [18], whereby specific DNA was not detected. Additionally, an ophthalmic examination was performed on each cat; eyelash and cartilage abnormalities and incorrect positioning of the eyelids were ruled out. Irregularities of the drainage system were eliminated with a 1% fluorescein test and by irrigation via a 26 G catheter.

Swabs were taken prior to any other ocular examination and transferred to the bacteriological laboratory at the Department of Epizootiology, Faculty of Veterinary Medicine, Wrocław. Material from the swabs was suspended in 500 *μ*L of 0.9% NaCl by vortexing for 2 minutes. DNA was extracted from material released from the ocular swabs using the QIAamp UltraSens Virus Kit (Qiagen, Syngen Biotech, Wrocław, Poland), in accordance with the manufacturer's instructions. The quantity of DNA was measured using NanoDrop 2000. Amplification of the conserved region within eubacterial 16S rRNA gene was performed with primers 16S-27f AGAGTTTGATCMTGGCTCAG and 16S-907r CCGTCAATTCMTTTRAGTTT, yielding an 880 bp product. Next, nested PCR using 16S-27f primer and 16S-519r GWATTACCGCGGCKGCTG was performed, yielding a product of 492 bp (all primers were from http://www.ridom.de/rdna/). PCR was performed under the following conditions: initial denaturation at 94°C for 3 min, followed by 35 cycles of 94°C for 30 s, 54°C for 45 s, and 72°C for 30 s. The 492 bp PCR products were ligated into the pJET1.2/blunt cloning vector (Thermo Scientific). Laboratory* E. coli *NovaBlue strain (Novagen) was transformed with a ligation product using heat shock, and the cells were plated onto agar containing ampicillin. Positive clones with amplicon-containing vectors were PCR-amplified using pJET1.2 sequencing primers and sequenced. The sequences obtained from both strands of the PCR product were analyzed using BioEdit software (http://www.mbio.ncsu.edu/BioEdit/bioedit.html) and taxon identification was conducted using the https://blast.ncbi.nlm.nih.gov platform. Taxa were identified at a species level when the similarity of their 16S rRNA sequence and those from the GenBank database were >99%. Sequence similarity between 97 and 99% was the criterion for identification of the taxon at the genus level.

## 3. Results

**Figure 1:**
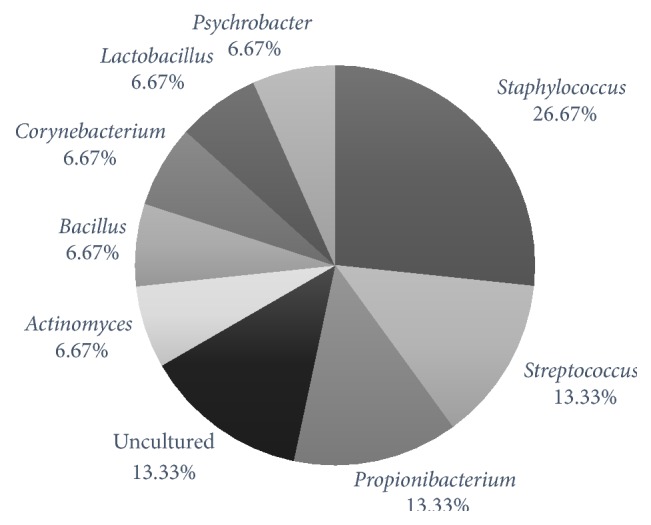
Diversity of bacterial genera identified within feline conjunctivas, based on a 16S rRNA gene sequence analysis.

A total of 48 sequence reads were obtained in the study; only the 30 high-quality sequence reads were used in further analysis of the diversity of bacterial flora in the feline conjunctiva. Eight genera were identified among the sequences from clinically healthy and diseased animals (Figure 1). Taking into consideration the maximal 16S rRNA distance scores < 1%, the following species were recognized:* Bacillus subtilis*,* Psychrobacter faecalis, Psychrobacter pulmonis, Propionibacterium acnes, Staphylococcus caprae, Staphylococcus capitis, Staphylococcus succinus, Streptococcus infantarius*, and* Streptococcus lutetiensis*. The low similarity in microflora composition at the genus level was observed between diseased and healthy conjunctivas (Table 1).

## 4. Discussion

The limited capacity of culture-based methods for the identification of bacteria from the feline conjunctiva makes standard procedures incomplete. This is mainly due to the limited viability of some microbial species, coinfections, or the presence of uncultivable or as yet unknown species. The monitoring of feline conjunctiva using alternative methods is not commonly applied as a standard for analyzing the diversity of conjunctival microflora in cats. DNA-based approaches were already used to assess the diversity of microbial communities or to monitor population dynamics [16]. The analysis of bacterial taxa in conjunctival swabs by DNA sequencing provided evidence that feline conjunctiva may be settled by microorganisms not yet isolated. Our results, compared with those of culture-based studies, suggest that the diversity of bacterial flora within feline conjunctiva can vary more than previously believed. We found that our results based on sequence analysis methods were concordant with the culture-based analysis previously applied to the same material in terms of genera such as* Bacillus* sp.,* Staphylococcus* sp., and* Streptococcus* sp. [19]. Bacteria belonging to these genera had already been identified in cat conjunctivas [2–5]. A comparison of eye microflora of clinically healthy animals and those with signs of conjunctivitis indicated no qualitative differences [19]. The results of our study revealed some species that had not been reported earlier in feline conjunctiva, including* Bacillus subtilis*,* Staphylococcus caprae*,* Staphylococcus succinus, Streptococcus infantarius, Streptococcus lutetiensis, Psychrobacter faecalis*, and* Propionibacterium acnes*.

*Psychrobacter* sp. belongs to the gamma Proteobacteria family and includes bacteria isolated from the skin of fish and chickens, meat products, clinical sources, and sea water [20]. In our study, bacteria from* Psychrobacter* taxon constituted a considerable subpopulation.* Psychrobacter faecalis* is a new species, isolated from pigeon feces and from human samples [21, 22]. In 2003, some human species previously identified as* Psychrobacter immobilis* were reevaluated and assigned to the species* P. faecalis*. Gini [23] described an ocular infection acquired in hospital caused by* Psychrobacter immobilis*. In the present study, one sequence also showed a similarity to* Psychrobacter pulmonis*, a novel subline within the genus* Psychrobacter*, isolated previously from lambs and humans [19, 24].* Staphylococcus succinus* and* Staphylococcus caprae* belong to the coagulase-negative staphylococci (CNS). They may colonize the skin surface and mucous membranes of mammals. To the best of our knowledge, there were no reports on the isolation of these species from cat conjunctivas.* S. caprae* was originally associated with goats and identified as an etiologic agent of intramammary infections [25, 26]. These bacteria were also detected in humans with bloodstream, urinary tract, bone, and joint infections as well as a commensal on human skin [27].* S. succinus* ubiquitously occurs in the environment [28], but it was also isolated from clinical samples (pus, blood, CSF, exudates, eye swab, or wound swab) from humans with various clinical disorders [29].

*Lactobacillus salivarius* was isolated from the gastrointestinal tract and oral cavity of hamsters and from the intestinal tract of swine and chickens [30]. It serves as a common component of probiotic substances. Thus, its presence in cat conjunctivas can be explained by nursing cats with milk products.

It was shown that the 16S rRNA gene sequencing can have low strength for the discrimination of species in the genus* Bacillus* [31]. In our study, the 16S rRNA gene sequence homology to* Bacillus subtilis* was 100%. Moreover, isolates assigned to* Bacillus* were previously identified in cats' conjunctivas in our laboratory by microbiological means [19].

Three genera belonging to Actinobacteria were found only in cats with signs of conjunctivitis. It was shown that* Propionibacterium, Actinomyces*, and* Corynebacterium* may constitute commensal and environmental bacterial flora and could be acquired by pets from human.* Propionibacterium acnes* is an example of a bacterium of human origin, frequently considered a commensal colonizer of human skin, one which is involved in inflamed acne breakouts [32]. Although* Corynebacterium*, also referred to as diphtheroids, are considered nonpathogenic, they have been recognized as the cause of serious systemic and ocular infections. Bacteria belonging to* Corynebacterium* are associated with conjunctivitis, keratitis, and endophthalmitis in humans [33]. Actinomycosis in cats is typically related to the oropharyngeal, thoracic, or abdominal cavity infection and associated with the migration of plant foreign bodies [34].* Actinomyces *spp. were most frequently isolated from cat pyothorax and subcutaneous wounds [35]. Their role in feline conjunctivitis was not recognized; nevertheless, the bacteria can grow in anaerobic or facultatively anaerobic environment, which can also be found in conjunctival sacs. Concurrent or prior multiplication of facultative aerobic bacteria in tissues may also decrease the oxygen level, creating an environment supporting anaerobic bacteria growth [36].

## 5. Conclusion

The feline conjunctiva may be inhabited by a diverse microbial community consisting of hundreds or thousands of species, with relatively few genera predominating. Our study demonstrates that the feline conjunctival sacs are inhabited by a much more rich and diverse microbial community than could be inferred from culture-based methods. Feline conjunctivas could also be colonized with unculturable bacteria, which limits their standard diagnostics. In this case, demonstration of such species in the diagnostic context may constitute a new area for research on the etiology of feline conjunctivitis. In our opinion, it is worth focusing on the bacteria, which could be overlooked during a standard bacteriological investigation, for example, as detected in our study, actinomycetes which require customized incubation time (longer than 7 days). Furthermore, they belong to the leading producers of substances showing biological activity, which could interfere with a selection of antibiotic-resistant strains of other bacteria. As yet, the role of actinomycetes in feline conjunctivitis has not been established, but it is clear that other standards for cultivation or examination targeted at molecular detection should be taken into account. Clinical relevance of these microbiota requires further study.

## Figures and Tables

**Table 1:** Bacterial genera identified in diseased and clinically healthy cat conjunctiva.

	Healthy cats			Cats with conjunctivitis		
	1	2	3	1	2	3
*Actinomyces *spp.				*∗*		
*Bacillus *spp.			*∗*			
*Bacillus subtilis*			*∗*			
*Corynebacterium *spp.					*∗∗*	
*Lactobacillus *spp.	*∗∗*					
*Lactobacillus salivarius*	*∗∗*					
*Staphylococcus *spp.		*∗*				*∗*
*Staphylococcus capitis*		*∗*				
*Staphylococcus caprae*		*∗*				
*Staphylococcus pasteuri*						*∗*
*Staphylococcus succinus*	*∗*		*∗∗*			
*Staphylococcus warneri*						*∗*
*Streptococcus infantarius *		*∗*				
*Streptococcus lutetiensis*		*∗*				
*Propionibacterium *spp.				*∗*	*∗∗*	
*Propionibacterium acnes*				*∗*	*∗∗*	
*Psychrobacter *spp.	*∗*					
*Psychrobacter faecalis*	*∗*					
*Psychrobacter pulmonis*	*∗∗*					
Uncultured bacterium	*∗*	*∗*			*∗∗*	

^*∗*^>99% identity to sequences from GenBank; ^*∗∗*^an identity between 97 and 99% to sequences from GenBank.
